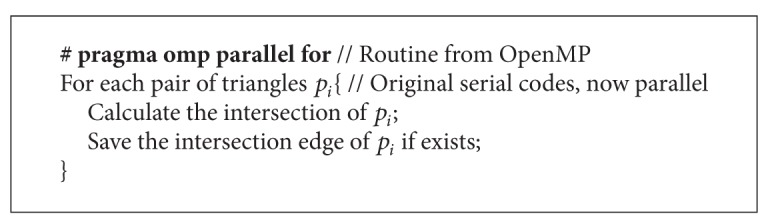# Summary on Several Key Techniques in 3D Geological Modeling

**DOI:** 10.1155/2014/723832

**Published:** 2014-02-16

**Authors:** Gang Mei

**Affiliations:** Institute of Earth and Environmental Science, University of Freiburg, Albertstr. 23B, 79104 Freiburg im Breisgau, Germany

## Abstract

Several key techniques in 3D geological modeling including planar mesh generation, spatial interpolation, and surface intersection are summarized in this paper. Note that these techniques are generic and widely used in various applications but play a key role in 3D geological modeling. There are two essential procedures in 3D geological modeling: the first is the simulation of geological interfaces using geometric surfaces and the second is the building of geological objects by means of various geometric computations such as the intersection of surfaces. Discrete geometric surfaces that represent geological interfaces can be generated by creating planar meshes first and then spatially interpolating; those surfaces intersect and then form volumes that represent three-dimensional geological objects such as rock bodies. In this paper, the most commonly used algorithms of the key techniques in 3D geological modeling are summarized.

## 1. Introduction

“Geological modelling is the applied science of creating computerized representations of portions of the Earth's crust based on geophysical and geological observations made on and below the Earth surface [1].” Mallet [2] defined the geological modeling as a collection of mathematical approaches for simulating the topological, geometrical, and physical properties of geological objects. Houlding [3] introduced some basic methods of geological modeling including the spatial data analysis, geological interface modeling, and geological boundary connection.

There are two essential procedures in 3D geological modeling: (1) the modeling of geological interfaces and (2) the building of three-dimensional geological objects. Usually, the geological interfaces are represented by geometric surfaces; these geometric surfaces can be mathematically parametric such as NURBS [4, 5] and Bézier [6] or discrete such as polygonal surface meshes [7, 8]. Both of the kinds of surfaces are widely used to model and simulate geological interfaces. More details can be found in the literature [4–11].

When adopting discrete surfaces to represent geological interfaces, the surface meshes can be generated directly in 3D (i.e., the surface mesh generation) [11], or alternatively, obtained by creating planar meshes first and then interpolating into 3D [8, 9]. The first method allows modelers to simulate quite complex geological interfaces, but costs much more efforts to generate surface meshes. The second is relatively easier to implement, especially when the geological interfaces are layered; in this method, mesh generation algorithms are accepted to create planar meshes, and interpolation algorithms are used to transform the polygonal meshes from 2D to 3D. In this paper, we summarize such algorithms of mesh generation and spatial interpolation.

Three-dimensional geological objects such as rock bodies can be represented by wire-frames, voxels (e.g., tetrahedron, prism, and hexahedron), and boundaries (i.e., sets of mathematically parametric surfaces or discrete surface meshes). When represented by discrete surface meshes, the three-dimensional geological objects are exactly the volumes bounded with polygonal meshes. Generally, such volumes that represent geological objects are created by the intersection of surface meshes [2, 7, 8]. Of the surface meshes, the triangulated surface is the most widely used. This paper also describes the intersection of triangulated surfaces.

The rest of this paper is organized as follows. The most commonly used algorithms in the planar mesh generation and spatial interpolation are summarized in Sections 2 and 3, respectively; and then, the intersection of triangulated surfaces is briefly introduced in Section 4; finally, we conclude this summary in Section 5.

## 2. Planar Mesh Generation

Many algorithms for generating planar meshes have been proposed, including the Delaunay triangulation method [12], advancing front technique (AFT) [13], ear-cutting [14], and the greedy algorithm [15]; see [16, 17] for surveys. Among them, the first one of the most popularly used is the Delaunay triangulation algorithm which intends to create high quality elements with biggest smallest angles by nature, and the second one perhaps is the advancing front technique although it does not have so solid mathematical foundations as that of the Delaunay triangulation method. In this section, the Delaunay triangulation method including the standard form and the constrained form, the advancing front technique will be described. In addition, several algorithms proposed for dividing polygons [18, 19] or for improving quality of meshes are also introduced.

### 2.1. Delaunay-Based Triangulation

The *Delaunay triangulation* (DT) is the straight-line dual structure of the *Voronoi diagram*; see [20] for the clear definitions of the Delaunay triangulation and constrained Delaunay triangulation. The Voronoi diagram is a type of geometric structure that can be used to represent the proximity relationships for a set of sites/points. The Voronoi diagram was first presented by Dirichlet in 1850 [21] and developed in further by Voronoi [22] in 1908.

Voronoi diagrams have been successfully used in various applications such as nearest neighbor search, facility location, largest empty circle, robot navigation/path planning, and high quality triangulation [23]. Many algorithms have been designed to construct Voronoi diagrams [24, 25]; see surveys in [26, 27]. A commonly used one is the incremental algorithm which inserts a new point/site into a previous existing diagram sequentially until no sites left. The sweep line algorithm proposed by Fortune [28] is more efficient in time than other incremental algorithms.

The Delaunay triangulation method is currently the most popular generic mesh generation method. There are various algorithms proposed for constructing DTs, such as divide-and-conquer method [29], sweep line [28], and incremental algorithm [30]. The basic idea behind the incremental algorithms is to insert points into an existing triangulation one by one and then modify some local triangles. The incremental algorithm was thought initially proposed in 1977 by Lawson [30], and later, Bowyer [31] and Watson [32] developed it further in 1981. The above two algorithms referred as the *Lawson algorithm* and the *Bowyer-Watson algorithm* are the most widely used Delaunay triangulation algorithms.

In the standard Delaunay triangulation algorithm, the input for constructing DTs is only a set of discrete points. All needed to do in the Delaunay triangulation algorithm is to form finite Delaunay triangles by using all given points as the required triangles' vertices. However, in applications, the input for triangulation is usually not only composed of discrete points but also some fixed segments or faces (in 3D). Those segments or faces are considered as constraints because they must be respected in triangulations.

When the input for Delaunay triangulation no longer consists of only points but points and segments in the plane, the algorithm that constructs triangulation which is as close as possible to the standard Delaunay triangulation is called *constrained Delaunay algorithm*; and the triangulation generated by the constrained Delaunay algorithm is the *constrained Delaunay triangulation* (CDT) [20, 33].

The constrained Delaunay triangulation is a generalized form of the Delaunay triangulation which forces some constrained segments into the resulting triangulation. Noticeably, due to introducing the constrained segments, often a CDT that contains certain required segments/edges does not meet the Delaunay condition but will be as close as possible to that of the standard DT [20, 33].

The algorithms for constructing DTs can be also improved to create CDTs, such as divide-and-conquer [20], sweep line [34] and incremental algorithm [35]. Most of them run in worst-case *O*(*n*log⁡*n*) time, but the most popularly used method for generation CDTs is the incremental insertion. The idea behind incremental algorithm for CDT is similar to that for DT: firstly without considering the constrained segments, a Delaunay triangulation is created for the PSLG's vertices using any type of standard Delaunay algorithm; and secondly the constrained segments are inserted into the existing triangulation one by one, and several local triangles need to be updated to satisfy the Delaunay property as close as possible after each insertion. A quite robust and fast framework for constructing the CDTs is the package TRIANGLE [35].

### 2.2. Advancing Front Technique (AFT)

The advancing front technique (AFT) is one of the most popular approaches for generating triangulations. The classical form of the AFT for mesh generation was described by [13, 36] and some improved versions of the classical form have been developed; see [37–40]. The AFT has become a successful method for creating high quality unstructured triangular or tetrahedral meshes for domains of arbitrary shape. In addition, it has been extended to generate quadrilateral meshes in 2D and hexahedral meshes in 3D; see [41–43].

**Figure 1:**
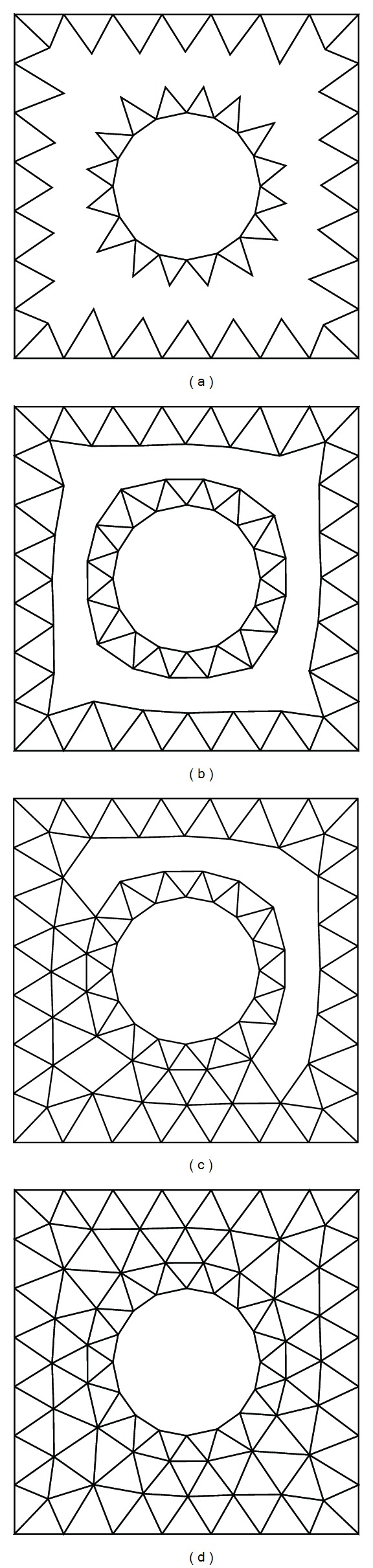
The AFT for generating planar triangulations.

*General Scheme.* The AFT is implemented based on the updating (advancing) of the so-called *front*; the front is a set of edges in 2D or a set of faces in 3D. The AFT can be considered as an iterative procedure that starts from the discretization of a given boundary and then intends to mesh those currently unmeshed region of the target domain with some type of specific elements such as triangles or tetrahedrons. A simple application example is presented in Figure 1 to illustrate the procedure of the AFT approach.

In the classical AFT, the field points that will be used as the candidates of the optimal vertices for those selected front entities are created synchronously when needed, rather than before creating any mesh element (i.e., preplaced interior points). Based on the classical AFT, many variants have been presented; see [44] for a survey.

The inherent procedure in all AFT-based algorithms is the local inserting and updating according to some type of geometric criteria. In the classical AFT, the creations of mesh vertices and elements are synchronous. However, in some variants of the AFT, the interior points that will be adopted as the mesh vertices are precreated before forming mesh elements; and when creating new elements, the optimal vertices are chosen from the existing interior points rather than created when needed.

The most important feature of the AFT approach is that it intends to create high quality elements and well-graded meshes, especially near the boundary of mesh domain. The feature of nicely respecting to the domain boundary is quite useful for generating meshes for the domain with very complex boundaries. The main disadvantage of the AFT approach is its convergence: compared to the Delaunay-based algorithms, the AFT does not have the sold mathematical foundations and cannot be proved to be always convergent strictly in mathematics.

### 2.3. Polygon Triangulation

Polygon partitioning is the procedure of decomposing a polygonal area into simpler components such as triangles [45, 46], trapezoids [47], or even subpolygons [48]. Usually, polygons are divided into sets of triangles; and this partitioning is also referred to as *Polygon Triangulation*.

A family of the most commonly used algorithms for triangulating polygons is the ear-cutting method. Removing an ear results in forming a new polygon that still meets the “two ears” condition (proved by Meisters [14]) and repetitions can be done until there is only one triangle left. This is known as the *ear-clipping* or *ear-cutting* algorithm.

The ear-clipping triangulation algorithm consists of searching an ear and then cutting it off from current polygon. The original version of Meisters's ear-clipping algorithm runs in *O*(*n* ^3^) time, with *O*(*n*) time spent on checking whether a newly formed triangle is valid. Rourke [18] simply modified and reorganized Meisters's algorithm and made the new version of ear-clipping algorithm run in *O*(*n* ^2^) time. An efficient technique named “prune-and-search” for finding an ear in the linear time was discovered by ElGindy [49]. Held [50] designed a completely reliable triangulation algorithm and engineered its code FIST.

**Figure 2:**
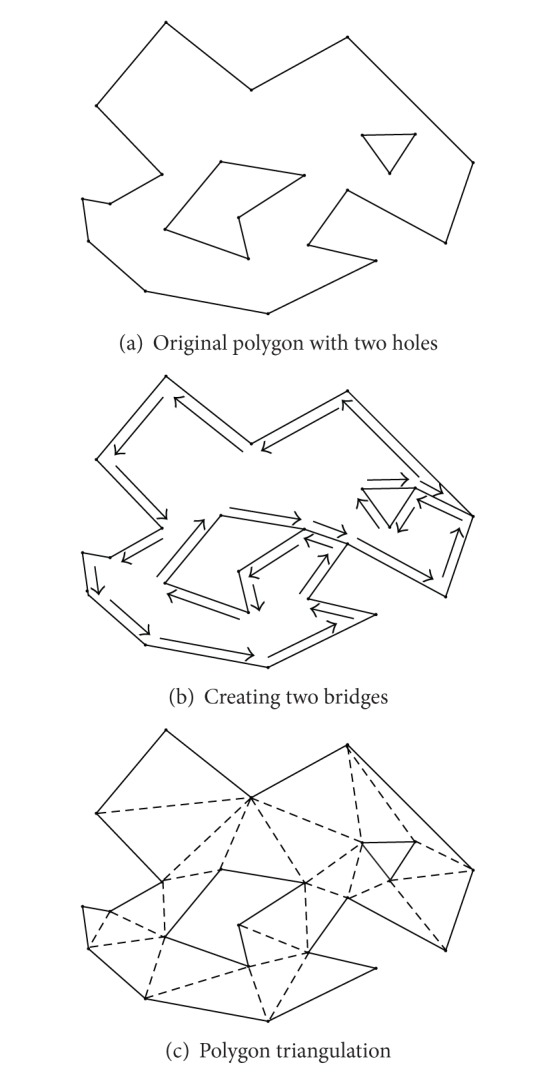
Creating bridges and triangulation for the polygon with holes [51].

The generic ear-cutting algorithms are only capable of triangulating simple polygons that have no holes. When polygons have holes (multiply connected polygonal area), a preprocessing of creating “Bridge” edges needs to be done in order to transform the polygon with holes into a single polygon. After transforming a polygon with holes to a degenerate polygon, the ear-cutting algorithms can be accepted to generate the final triangulation; see Figure 2. Noticeably, some vertices can be accepted to form triangle twice due to introducing those “Bridge” edges.

Creating “Bridge” edges is widely used to divide a general closed domain into several simply connected, convex subdomains; and Delaunay triangulations for subdomains then are obtained in turn and patched together to generate the whole triangulation or then its dual graph, Voronoi diagram; see Tipper [25]. Similarly, Held [50] adopted “Bridge” edges to transform a multiply connected polygonal area into a single polygon which can be triangulated by ear-clipping based algorithms.

### 2.4. Mesh Smoothing

Usually, a mesh needs to be optimized after being newly created to improve its quality, and various methods have been developed to deal with this issue. Mesh smoothing is the method for improving the mesh quality by adjusting the positions of mesh vertices without altering the topology of the mesh. The basic idea behind mesh smoothing is to make all elements in a mesh be their best shapes by repositioning the vertices' coordinates.

Numerous papers have been published on the topic of mesh smoothing. In the literature [52], Mei et al. introduced a useful classification to divide the methods of mesh smoothing into four types: (1) geometry-based methods, (2) optimization-based methods, (3) physics-based methods, and (4) hybrid methods.

Geometry-based mesh smoothing methods obtain new node locations either by using geometric rules or local optimization techniques or by minimizing objective functions. The most popular geometry-based method is Laplacian smoothing [53], which at each iteration step repositions each node at the centroid of its neighboring nodes. To improve the performance of its basic form, some smart, constrained or weighted variations have been proposed [54–56].

Other simple or effective geometry-based methods include the angle-based approach [57], the geometric element transformation method (GETMe) [58], a projecting/smoothing method [59], an algebraic method [60], the target-matrix paradigm [61], the effective variational method [62], and a novel method based on quadric surface fitting, vertex projecting, curvature estimating, and mesh labeling [63].

Optimization-based methods obtain the smoothed node positions by minimizing some given distortion metric. These methods give better results than most of the geometry-based methods, especially in concave regions; however, they are computationally more expensive. Some of the optimization-based methods are described in [64–66].

Physics-based mesh smoothing methods operate by physical processing [67] or by solving simple physics problems. For instance, Shimada et al. [68] proposed a method which treats nodes as the centers of bubbles and obtained the smoothed node locations by deforming these bubbles against each other. A similar algorithm, the pliant method, is presented in [69].

Hybrid mesh smoothing methods combine two or more basic methods; this is for the purpose of improving the performance of each. Some of them are the combinations of Laplacian smoothing with various optimization-based methods [70–72].

The most commonly used mesh smoothing method is Laplacian smoothing, which in its original form [53] moves each node to be at the average of all the nodes connected to it by element edges. Laplacian smoothing is an iterative algorithm; the iterations will repeat until there are no points that are moved in the same iteration step beyond a given distance tolerance. Several modifications and enhancements of this method have been described in [54, 72]. A noniterative method called Direct Method was introduced by Balendran [73]. An iterative modification of the Direct Method, the Modified Direct Method (MDM), is presented in [74].

## 3. Interpolation Algorithms

The interpolation is the method of obtaining the evaluation value at an unknown point according to a set of known data points based on some types of relationships. In mathematics, there are various kinds of interpolation approaches such as linear interpolation, polynomial interpolation, spline interpolation, trilinear interpolation, and Gaussian interpolation [75]. In geosciences, the most popular interpolation methods perhaps are the following three: Kriging interpolation [76], Discrete Smooth Interpolation (DSI) [77, 78], and Inverse Distance Weighted (IDW) [79]. In this section, these three methods will be briefly summarized.

### 3.1. Kriging Method

In three-dimensional geological modeling, Kriging is usually adopted to generate guaranteed geological interfaces with effectively conforming to the original data and does not depend on existing meshes.

Kriging interpolation method [76] is an optimal geostatistical estimator for the regionalized variables within a limited area based on the variogram or covariance. The Kriging method was originally developed by the South African mining engineer Krige [80, 81]. The mathematics was further developed by Matheron [82] in 1963. More details about the Kriging method can be found in [83].

For a set of regionalized variables *Z*(*x*), Kriging estimates the expected value *Z*(*x*_0_) at the location *x*_0_ where the observation is not available by using a linear weighted sum of the known values *Z*(*x*_1_), *Z*(*x*_2_),…, *Z*(*x*_*n*_) at locations *x*_1_, *x*_2_,…, *x*_*n*_, such that

(1) $$\begin{array}{c} Z\left(x_{0}\right)=\overset{n}{\underset{i=1}{\sum }}\lambda_{i}\cdot Z\left(x_{i}\right),\\ \overset{n}{\underset{i=1}{\sum }}\lambda_{i}=1, \end{array}$$

where *λ*_*i*_ is the weights at the location *x*_*i*_ which can be calculated according to the system of equations of the Kriging.

The estimator of the Kriging is in fact the calculation of a linear weighted sum. Thus, the objective in Kriging method is to determine those weights *λ*_*i*_  (*i* = 0,1,…, *n*). There are two widely used estimators of the Kriging method: the *Ordinary Kriging* (OK) and *Universal Kriging* (UK). Both of them estimate the interpolation values with a linear weighted sum (1). The differences of them are the calculation of the weights *λ*_*i*_ in different ways due to different assumptions. The Ordinary Kriging assumes that the distribution of the observations meets the second order stationary condition; in other words, the mean of all input sample points is a constant value. The Universal Kriging assumes that the mean is no longer constant but as a polynomial function referred as the *drift* or *trend*.

### 3.2. Inverse Distance Weighted (IDW)

IDW (Inverse Distance Weighted/Weighting) [79] is a very simple and widely used geospatial interpolation method. The expected value of an interpolated point (unknown point) is the weighted average of all (or sometimes part of) the sample points. The weights only depend on the distances between the interpolation points and the sample points. A general form of finding an interpolated value *Z* at a given point *x* based on samples *Z*_*i*_ = *Z*(*x*_*i*_) for *i* = 1, 2,…, *n* via the IDW can be represented with the following formula:

(2) $$\begin{array}{c} Z\left(x\right)=\overset{n}{\underset{i=1}{\sum }}\frac{\omega_{i}\left(x\right)z_{i}}{\sum_{j=1}^{n}\omega_{j}\left(x\right)},\;\omega_{i}\left(x\right)=\frac{1}{d{\left(x,x_{i}\right)}^{p}}. \end{array}$$

The above equations are the simple IDW weighting functions defined by Shepard [79]. In the equations, *x* denotes an interpolated point, *x*_*i*_ is a sample point, *d* is the distance from the known point *x*_*i*_ to the unknown point *x*, *n* is the total number of known points used in interpolation, and *p* is a positive real number called the power parameter.

In contrast to Kriging, IDW also obtains the expected values of unknown points (interpolated points) by weighting average of the values of known points (data points). The name, that is, Inverse Distance, of this method was motivated by the weighted average applied since it resorts to the inverse of the distance to each known point when calculating the weights. The difference between IDW and Kriging is that they calculate the weights by different means.

In Kriging, it is needed to calculate the spatial correlations between (1) all sample points and (2) the interpolated points with the sample points and the spatial correlations that are expressed via the *Variogram* or *Covariance* depend on the relative positions of points rather than the distances between points.

In the IDW, however, the weights and correlations are only involved with the distances between points; two distinct sample points that have the identical distance to a same interpolated point definitely have identical weights. This approach of determining the weights only depending on distance is reasonable in some cases such as analyzing the propagation of sound or the spread of pollutants. But in other cases, for example, reconstructing the ground surface or creating DEM (Digital Elevation Model), the IDW is not as effective as the Kriging.

### 3.3. Discrete Smooth Interpolation (DSI)

The Discrete Smooth Interpolation (DSI) is an interpolation algorithm proposed by Mallet [78], which has become one of the key techniques in the geological modeling software GOCAD [84]. The basic ideas behind DSI are as follows: for those known and unknown nodes on a “grid” of a discrete natural object, the values attached to the unknown nodes can be obtained by making the unknown nodes satisfy the constraints (e.g., the roughness criterions) defined by the known nodes. This interpolation method can be applied in any dimension since it relies on the topology of nodes on a “grid.” More details about the DSI can be found in [2].

## 4. Intersection of Triangulated Surfaces

The Boolean operation (including the intersecting) of triangular surface meshes is currently well studied. The algorithms for calculating the intersection of triangulated surfaces have been widely used in the field of CAD/CAE/CAM; these algorithms can be also used to build three-dimensional geological bodies.

Lindenbeck et al. [85] developed the TRICUT package by coupling two reliable open source programs, the RAPID library [86] for collision detection and the TRIANGLE library [35] for robust constrained Delaunay triangulation to cut intersecting triangle meshes. Shostko et al. [87] introduced an algorithm that constructs surface meshes from a given set of intersecting triangulated surfaces. Lo [88] proposed an algorithm for determining the intersection lines based on tracing the neighbours of intersecting triangles when calculating the intersection of triangulated surfaces. Guo et al. [89] gave an algorithm to implement the Boolean operation of two STL solids based on tracing the intersection line segments to form loops. Pavi$\overset{´}{\text{c}}$ et al. [90] developed a hybrid method which combines polygonal and volumetric computations and representations for performing Boolean operations over polygonal meshes. Wang [91] presented a new approach to compute the approximate Boolean operations of two freeform polygonal mesh solids efficiently with the help of Layered Depth Images (LDIs).

Zhao et al. [92] adopted NVIDIA's CUDA [12] computing environment to develop an efficient, parallel, and scalable framework for evaluating approximate Boolean operations on polygonal meshes. A general algorithm for computing the intersection of manifold surfaces was presented in [93], which relies on a comprehensive list of edge-triangle intersection cases combined with an intersection tracking algorithm that utilizes both topological and geometrical consistency checks. Karamete et al. [94] used elementary computational-geometry operations such as, facet-segment intersection, point containment in simplices, and edge recovery in a plane, to produce high-level Boolean operations. Mei and Tipper [95] proposed a novel technique instead of inside/outside classification to distinguish the resulting union, subtraction, and intersection parts of intersected triangular surface meshes.

In calculating the intersection of triangulated surfaces, the intersection of a pair of triangles is the essential basis for the further operations such as tracing intersection lines (loops) and clipping the intersected surfaces. When computing the intersections for large and complex models composed of plenty of triangles, solutions are needed to speed up the procedure of intersecting. The following summarizes popular approaches for addressing two problems: (1) how to calculate the intersection of triangles fast and (2) how to speed up the intersection of surfaces effectively.

### 4.1. Triangle-Triangle Intersection Algorithm

Several quite efficient approaches have been proposed to calculate the intersection of a pair of triangles in 3D, including Möller's algorithm [96], Held's algorithm [97], Devillers and Guigue's algorithm [98, 99], Tropp's algorithm [100], and Shen's algorithm [101].

**Figure 3:**
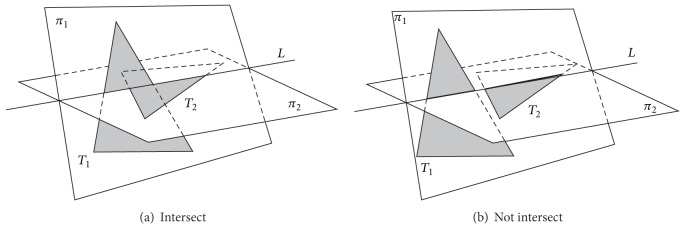
Intersection of triangle with triangle [96].

*Möller's Algorithm.* Möller [96] proposed a method named “interval overlap” for checking whether a pair of triangles intersects (Figure 3). The basic idea behind Möller's algorithm is that if the degenerate case that there is a triangle such as *T*_1_ whose three vertices completely locate on one side of the underlying plane of the other triangle *T*_2_ is rejected, then the intersection of the two underlying planes of *T*_1_ and *T*_2_ denoted as a line *L* will intersect *T*_1_ and *T*_2_. The intersections of the line *L* with the triangles *T*_1_ and *T*_2_ are two line segments (also called *intervals*). If those intervals overlap, then the triangles *T*_1_ and *T*_2_ intersect, otherwise, not intersect. 

*Held's Algorithm.* Very similar to the Möller's algorithm, the Held's algorithm [97] is also carried out via dimension deduction. However, in Möller's algorithm, the determination of intersection of two triangles in 3D is deduced to compute the overlap of two line segments in 1D; while in Held's algorithm, the calculation of the intersection of two triangles is transformed to determine the intersection of a line segment with a triangle in 2D. 

**Figure 4:**
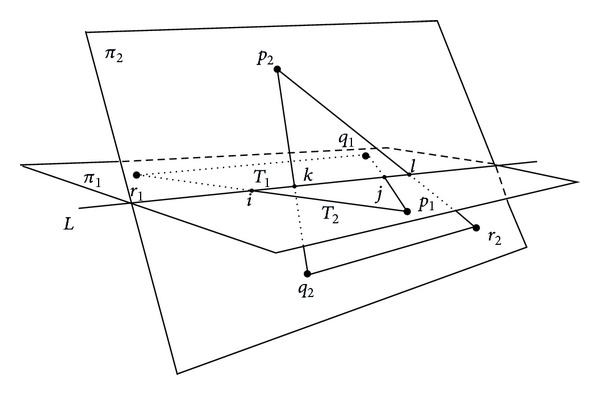
Computing the intersection of triangle-triangle with Devillers algorithm [98, 99].

*Devillers and Guigue's Algorithm.* In Devillers and Guigue's algorithm [98, 99], each vertex of each triangle is classified with respect to the other triangle using six orientation predicates: the triangle *T*_1_ is first tested for intersection with the plane *π*_2_ and the algorithm classifies the vertices of *T*_1_ with respect to the plane *π*_2_ by simply comparing the signs of the three determinants [*p*_2_, *q*_2_, *r*_2_, *p*_1_], [*p*_2_, *q*_2_, *r*_2_, *q*_1_], and [*p*_2_, *q*_2_, *r*_2_, *r*_1_]; see Figure 4. 

*Tropp's Algorithm.* Unlike Möller's or Held's algorithms, the Tropp's algorithm [100] intends to carry out the triangle-triangle intersection test in the algebraic viewpoint, rather than the geometric viewpoint. In the Tropp's algorithm, the process of determining the intersection of a pair of triangles is well speeded up by taking advantage of the linearity of arithmetic operations on relevant metrics and the strong linear relations between the columns of relevant metrics.

### 4.2. Accelerations for the Intersecting

The procedure of computing the intersections would be quite slow when there are a huge number of triangles of the surface meshes. Accelerations need to be carried out to improve the efficiency. Generally, there are two commonly used strategies: the first is to reduce the intersection tests (also called *collision detection* tests) of triangles by using space partitioning techniques and the second is to implement the intersecting in parallel by taking advantage of those libraries designed for parallelization. In the following paragraphs, the above two strategies will be introduced.

#### 4.2.1. Speeding Up by Space Partitioning

**Figure 5:**
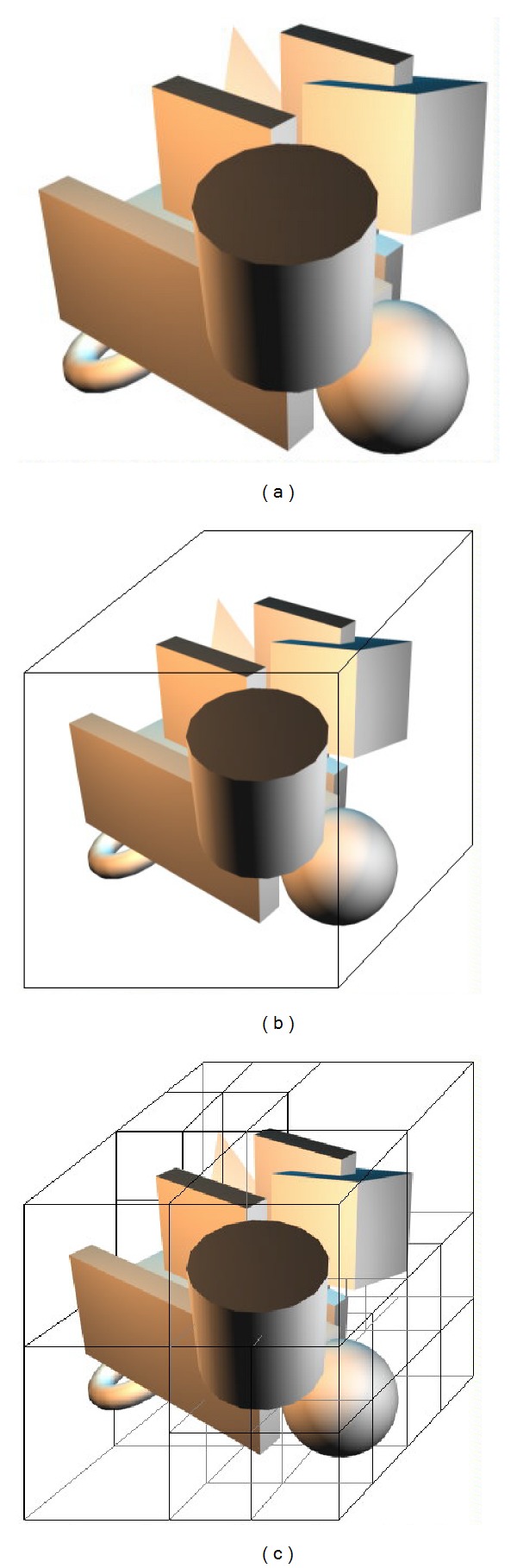
The creation of Octree [102].

The basic ideas behind space partitioning are as follows:

- space is divided into cells (i.e., the subspaces);
- object primitives are placed into cells (e.g., Octree); see Figure 5(b);
- object primitives within the same cell are checked for collision; see Figure 5(c);
- pairs of primitives that do not share the same cell are not tested.

Figure 5 shows a simple procedure of performing the space partitioning. The basic idea behind this strategy is easy to understand: a pair of triangles within the same subspace needed to check for intersection; and any pair of triangles that do not locate inside the same subspace is not needed to test. The objective of such procedure is to accurately find out all pairs of potentially intersected triangles in a short time to reduce computational cost. Many techniques based on space partitions such as BSP [103], Octree [90], OBB trees [86], AABB trees [104], and uniform grid [105] have been developed to realize this goal.

The easiest space partitioning is the dividing of space into a structured grid. Each element of the grid, that is, one of the subspaces, is a Cuboid or even a Cube. This method of partitioning is easy to realize in programming. The disadvantage of this kind of dividing is that plenty of subspaces are created; and the determination for checking which triangles locate within the same space is also time-consuming.

A better partitioning is by using Octree [102]; see Figure 5. The first step is to divide the target space into 8 subspaces, then putting the geometric objects such as triangles into those 8 subspaces and checking the number and density of the objects within the same subspace to decide whether it is needed to divide a subspace into 8 smaller subspaces. This procedure of dividing iterates until some kind of conditions or requirements are met. The advantage of Octree is that it is “smart” to decide whether it is needed to create the next level of subspaces for a previously created subspace. The disadvantage is that the establishment of Octree is more complicated than that of the grid.

#### 4.2.2. Speeding Up by Parallelization

The intersection of a pair of triangles does not affect the intersection of another pair. This is the precondition of conducting parallelization for computing the intersections. Hence, several pairs of triangles can be calculated in parallel to reduce the computational cost; this is the basic idea behind the strategy of speeding up the intersection of triangulated surfaces by parallelization.

There are many models designed for implementing parallelization; the four most frequently used are OpenMP [106], MPI [107], CUDA [108], and OpenCL [109]. OpenMP is an API that supports multiplatform shared memory multiprocessing programming. MPI (Message Passing Interface) is a library specification designed for high performance on both massively parallel machines and on workstation clusters. CUDA is a parallel computing platform and programming model, which enables increases in computing performance by harnessing the power of GPUs.

The algorithms for calculating the intersection of triangles, such as the Möller's algorithm [96], can be easily implemented within the popular parallelization models such as OpenMP or CUDA and become parallel and thus much more efficient in time when compared to their corresponding serial versions [92, 95]. The following piece of code is an example of implementing the parallelization from serial code to parallel based on OpenMP (see Algorithm 1).

## 5. Conclusion

We have summarized the most commonly used algorithms and approaches in mesh generation, spatial interpolation, and surface intersection. Those techniques are widely used when building 3D geological models. The most commonly used algorithms for generating triangular meshes in 2D, including the Delaunay-based, the AFT, and the ear-cutting, have been summarized. Also, the mesh smoothing algorithms for improving mesh quality are reviewed. These generic mesh generation algorithms are usually accepted to create planar meshes for modeling geological interfaces. We have also introduced three spatial interpolation algorithms, for example, the Kriging method, the DSI, and the IDW. In addition, one of the most important geometric computations, the intersection of triangulated surfaces, is summarized. We have listed the algorithms for calculating the intersection of a pair of triangles and explained how to speed up the intersection of triangulated surfaces according to the strategies of parallelization and space partitioning. A brief overview of several key techniques in 3D geological modeling is intended to present in this summary.

## Figures and Tables

**Algorithm 1:**